# Phloretamide Prevent Hepatic and Pancreatic Damage in Diabetic Male Rats by Modulating Nrf2 and NF-κB

**DOI:** 10.3390/nu15061456

**Published:** 2023-03-17

**Authors:** Rasha Al-Hussan, Nawal A. Albadr, Ghedeir M. Alshammari, Soheir A. Almasri, Mohammed Abdo Yahya

**Affiliations:** Department of Food Science and Nutrition, College of Food and Agricultural Sciences, King Saud University, Riyadh 11451, Saudi Arabia

**Keywords:** phloretamide, diabetes, NAFLD, Nrf2, NF-κB, rats

## Abstract

This study examined the effect of phloretamide, a metabolite of phloretin, on liver damage and steatosis in streptozotocin-induced diabetes mellitus (DM) in rats. Adult male rats were divided into two groups: control (nondiabetic) and STZ-treated rats, each of which was further treated orally with the vehicle phloretamide 100 mg or 200 mg. Treatments were conducted for 12 weeks. Phloretamide, at both doses, significantly attenuated STZ-mediated pancreatic β-cell damage, reduced fasting glucose, and stimulated fasting insulin levels in STZ-treated rats. It also increased the levels of hexokinase, which coincided with a significant reduction in glucose-6 phosphatase (G-6-Pase), and fructose-1,6-bisphosphatase 1 (PBP1) in the livers of these diabetic rats. Concomitantly, both doses of phloretamide reduced hepatic and serum levels of triglycerides (TGs) and cholesterol (CHOL), serum levels of low-density lipoprotein cholesterol (LDL-c), and hepatic ballooning. Furthermore, they reduced levels of lipid peroxidation, tumor necrosis factor-alpha (TNF-α), interleukin-6 (IL-6), mRNA, and total and nuclear levels of NF-κB p65, but increased mRNA levels, total and nuclear levels of Nrf2, as well as levels of reduced glutathione (GSH), superoxide dismutase (SOD-1), catalase (CAT), and heme-oxygenase-1 (HO-1) in the livers of diabetic rats. All of these effects were dose-dependent. In conclusion, phloretamide is a novel drug that could ameliorate DM-associated hepatic steatosis via its powerful antioxidant and anti-inflammatory effects. Mechanisms of protection involve improving the β-cell structure and hepatic insulin action, suppressing hepatic NF-κB, and stimulating hepatic Nrf2.

## 1. Introduction

Type-1 diabetes mellitus (DM) is the most common endocrine disorder that results from a deficiency of insulin, mainly due to autoimmune destruction of the pancreatic beta-cells [1]. The disease is associated with several co-morbidities that raise the mortality rates among affected individuals [2]. Nonalcoholic fatty liver disease (NAFLD) is the most common liver disease associated with T1DM [2]. The disease is caused mainly by metabolic disturbance, which leads to persistent hepatic de novo lipogenesis (DNL), impaired fatty acid (FA) oxidation, and sustained gluconeogenesis [3,4,5,6]. Although the disease is initiated as simple steatosis, an accumulative line of evidence has shown that locally induced oxidative stress, mediated by the overproduction of reactive oxygen species (ROS), is the leading mechanism responsible for the progression to non-alcoholic steatohepatitis (NASH) and liver injury by promoting inflammation, fibrosis, and apoptosis [7,8]. Major resources of such ROS in the livers of T1DM include endoplasmic reticulum (ER) stress, mitochondria dysfunction, activation of NADPH oxidase, and scavenging/reduced expression of endogenous antioxidants [7,8].

However, recent evidence has also shown that altered antioxidant signaling pathways contribute significantly to the development and progression of NAFLD in diabetic livers [9,10,11]. Among all, much focus has been given to the indispensable role of the nuclear factor erythroid 2-related factor 2 (Nrf2)/antioxidant axis in mediating and protecting against NAFLD in metabolically impaired and diabetic animals [12,13]. In general, Nrf2 is the major antioxidant transcription factor that stimulates cell survival by inducing phase II antioxidant enzymes such as heme oxygenase-1 1 (HO-1), catalase (CAT), superoxide dismutase (SOD), etc. [14]. In addition, Nrf2 has indirect antioxidant and anti-inflammatory effects and can increase insulin sensitivity by suppressing the nuclear factor kappa beta (NF-κB) [15,16]. In addition, experimental studies in HFD-fed animals have confirmed that Nrf2 can inhibit DNL and stimulate FA oxidation by promoting mitochondria biogenesis, suppressing the lipogenic sterol regulatory element-binding transcription factors (SREBPs), and activating FA oxidation [13,14,17,18]. In the cells, the transcriptional activity of Nrf2 is largely determined by a cytoplasmic inhibitor known as the Kelch-like ECH-associated protein 1 (Keap-1), which is tightly bound with Nrf2 [19,20]. In the presence of oxidative stress, ROS can modify keep-1, which results in the loss of this association and hence the nuclear translocation of Nrf2 [19,20]. The levels and nuclear activities of Nrf2 are significantly depleted in the livers of diabetic animals with T1DM and type 2 DM (T2DM), whereas the activation of this factor prevented oxidative liver injury by suppressing oxidative stress, inflammation, lipotoxicity, and apoptosis [9,10,11,20]. Hence, it seems reasonable that drugs that improve hepatic Nrf2 and antioxidant levels could protect against NAFLD [19,21].

Plant phytochemicals are natural activators for Nrf2, which has been largely studied for their potential to treat liver disorders [22,23]. Phloretin, a major flavonoid found in apple juice, has well-known anti-diabetic, hypoglycemic, hypolipidemic, antioxidant, and anti-inflammatory effects mediated mainly by activating the Nrf2/antioxidant axis [24,25,26]. Phloretamide [3-(p-Hydroxyphenyl) propionic acid] is a polyphenol derivative of phloretic acid, a major metabolite of phloretin metabolism, and acts as a growth hormone [27]. Until now, the pharmacological effects of phloetamide have not been well-characterized in rodents or animals, and such studies are still lacking. Interestingly, in a single old study, it has been reported that ex vivo treatment with phloretamide stimulates Nrf2 signaling in cultured hepatocytes [28]. Hence, it was worth targeting this molecule to characterize its potential to treat several metabolic and chronic disorders, which may provide a future novel therapy.

Streptozotocin (STZ) is the best-known commercial drug that leads to features of T1DM by damaging the majority of pancreatic β-cell and reducing serum insulin levels [29]. Therefore, in this study, we tested the hypothesis that phloretamide could alleviate NAFLD in SZT-induced diabetic rats by attenuating oxidative stress and inflammation. In addition, we have tested if this protection involves hypoglycemic and hypolipidemic effects and modulates the hepatic Nrf2/antioxidant axis.

## 2. Materials and Methods

### 2.1. Animals

Twelve-week-old male Wistar rats weighing 220–240 gm were obtained from the Experimental Animal Care Center, King Saud University (KSU), Riyadh, KSA. The animals were housed under environmentally controlled conditions (22 ± 5 °C, 55 ± 5% humidity) with a 12 h light/dark cycle. The rats were acclimatized to their environment for 2 weeks. During the entire period of the study, all animals had free access to water and chow and were housed in pairs in plastic cages. The diet used in this experiment was a normal standard control diet (cat # D12450B, Research Diets, Newbrunswick, NJ, USA) containing 10% fat (4.3 g%), 20% proteins (19.2 g%), and 70% carbohydrates (67.3 g%) with a total energy intake of 3.85 kcal/g). All experimental protocols included in this study were approved by the Research Ethics Committee (REC) at King Saudi University, Riyadh, Saudi Arabia, and all protocols were conducted according to the Animal Research: Reporting of In Vivo Experiments (ARRIVE) guidelines.

### 2.2. Establishment of DM

We have previously shown that Wistar rats are highly exposed to DM and significant depletion in insulin levels upon injection of STZ (50 mg/kg) [30]. This was the reason for selecting this species for this study. In accordance, DM was introduced to the rats of this study using a single intraperitoneal dose of STZ (50 mg/kg) which promotes an approximately 76% loss of pancreatic β-cells with a minimal mortality rate [29]. In brief, rats were fasted overnight and then injected with STZ (#75221, Sigma Aldrich, St. Louis, MO, USA) at the selected dose (prepared in 0.5 M sodium citrate) (pH = 7.4). Two weeks later, the rats fasted, and drops of whole blood were withdrawn from the rats’ tails and used to measure fasting glucose levels using a glucometer (Accu-Chek II Boehringer, ON, Canada). Those rats discovered to have blood glucose levels of >320 mg/dL were diagnosed with insulin deficiency DM. In addition, some of those rats were euthanized using neck dislocation, and their pancreases were collected and processed at the pathology laboratory for hematoxylin and eosin (H&E) staining to confirm the pancreatic damage.

### 2.3. Experimental Design

A total of 48 (control (24) and diabetic (24)) rats were selected randomly and divided into 6 groups (*n* = 8 rats/each) as follows: (1) control group: nondiabetic rats were orally administered the carrier, 0.5% low viscosity carboxymethylcellulose (CMC) (#5678, Sigma Aldrich, MO, USA); (2) phloretamide (100 mg/kg)-treated group: nondiabetic rats were orally treated with phloretamide dissolved in 0.5% CMC (phloretamide solution) at a dose of 100 mg/kg/day; (3) phloretamide (200 mg/kg)-treated group: nondiabetic rats were orally treated with phloretamide solution at a dose of 200 mg/kg/day; (4) STZ-diabetic model rats: diabetic rats were administered 0.5% CMC; (5) STZ + phloretamide (100 mg/kg)-treated group: rats with pre-established DM were orally treated with phloretamide solution at a dose of 100 mg/kg/day; (6) STZ+ phloretamide (200 mg/kg)-treated group: rats with pre-established DM were orally treated with phloretamide solution at a dose of 200 mg/kg/day. Phloretamide was synthesized and purchased from Yangzhou Chemical Co., Ltd., Jiangsu, China, based on previously published reports and structures [27,31,32]. All treatments were conducted for 12 weeks. Treatment with phloretamide solution was conducted by gavage using a special stainless-steel feeding cannula.

Food intake and body weight were monitored weekly. Dead rats in the diabetic groups were replaced with new rats. The selected doses of phloretamide were based on our preliminary data showing that 100 mg/kg was the minimum dose to reduce the fasting 24 h glucose levels in STZ-diabetic rats. In addition, we did not see any signs of renal, cardiac, or hepatic toxicities with these doses after 3 days of administration.

### 2.4. Serum Collection and Measurements

By the end of the treatment period, all rats were fasted overnight and were anesthetized with a low dose of ketamine/xylazine (50:5 mg/kg) [33]. In brief, rats were placed on a heated blanket, and their blood temperature was monitored using a digital rectal thermometer. All rats were kept normothermic throughout the anesthetic period. The anesthesia was confirmed when the rat lost its righting and toe withdrawal reflex. Blood samples were withdrawn from the heart of all anesthetized animals into EDTA or plain tubes, which were centrifuged at 3000 rpm to collect plasma and serum samples, respectively. These samples were stored at −20 °C and used later when needed.

### 2.5. Tissue Collection

After blood collection, all animals were euthanized using neck dislocation. Their livers were collected on ice, weighed, and washed with ice-cold phosphate-buffered saline (PBS, pH = 7.4). The liver of each rat was fractioned into smaller pieces, some of which were placed in 10% buffered formalin, and the others were snap-frozen in liquid nitrogen and then frozen at −80 °C until use. All formalin-preserved sections were processed within 20 h of collection at the pathology laboratory of KSU, Riyadh, KSA.

### 2.6. Analysis of the Serum and Plasma

The fasting glucose and insulin levels were assessed using commercially available kits formulated specifically for rats (#90010, Crystal Chem, Elk Grove Village, IL, USA and #DIGL-100, BioAssay Systems, Hayward, CA, USA) following each supplier’s instructions. In addition, we calculated the values from the homeostatic model assessment of β-cell function (HOMA-β) for each animal using the previously published equation [30]: HOMA-β = (Fasting insulin (ng/mL) × 20)/(Fasting glucose (mg/dL) − 3.5). Levels of total cholesterol (CHOL) and triglyceride (TG) serum levels were determined using multi-enzyme-based kits (#MBS726298, MyBioSource, San Diego, CA, USA, and #ECCH-100, BioAssay Systems, Hayward, CA, USA, respectively). Serum and hepatic levels of high-/low-density lipoprotein cholesterol (LDL-c/HDL-c were assayed using a special colorimetric kit (#E2HL-100 BioAssay Systems, CA, USA). Serum levels of alanine aminotransferase (ALT) and aspartate aminotransferase were analyzed using assay kits (#EALT-100 and EASTR-100, BioAssay Systems, CA, USA). All procedures were performed according to the manufacturer’s instructions for each kit (*n* = 8 samples/group).

### 2.7. Extraction of Lipid from Frozen Livers

This procedure followed the protocols published by our laboratories [30], which followed the method of Folch et al. [34]. Briefly, livers from each rat (125 mg) were soaked in a 2 mL methanol: chloroform mixture (1:2 *v*/*v*) for 4 h, followed by the addition of normal saline (0.25 mL). The whole mixture was centrifuged at 2000× *g* for 10 min. The lower layer was isolated, and the solvent was removed by evaporation in a rotatory evaporator. The dried lipids were then dissolved in 0.25 mL absolute isopropanol and then used while fresh for the measurement of lipid fraction via biochemical analysis using the same kits used to measure lipids in the serum and mentioned in the previous section above (*n* = 8 samples/group).

### 2.8. Preparation of the Tissue Homogenates and Nuclear Extraction

Liver samples (0.1 g) were homogenized in 9 volumes (0.9 mL) of lysis buffer consisting of 30 mM phosphate buffer and 140 mM KCl (pH = 7.3) at a ratio of 1:10, followed by centrifugation at 600× *g* for 10 min [35]. The supernatants were isolated, stored at 80 °C, and used later to measure antioxidants and inflammatory marker levels. The cytoplasmic/nuclear extracts were prepared using the nuclear extract kit (#40010, ActiveMotif, Tokyo, Japan). All procedures and measurements were conducted according to each manufacturer’s instructions.

### 2.9. Measurements of the Tissue Homogenates and Nuclear/Cytoplasmic Extracts

All kits used for this part were rat-specific. MDA ELISA kit for rats was used to measure levels of malondialdehyde (MDA) in the liver homogenates (#MBS268427, MyBioSource, CA, USA). GSH ELISA kit (#MBS265966; MyBioSource, CA, USA), SOD ELISA kit (#MBS036924, MyBioSource, CA, USA), CAT ELISA kit (#MBS726781, MyBioSource, CA, USA), IL-6 ELISA kit (#MBS269892, MyBioSource, CA, USA), HO-1 ELISA kit (MBS764989, MyBioSource, CA, USA) and TNF-αELISA kits (#MBS2507393, MyBioSource, CA, USA) were used to measure the levels of glutathione (GSH), superoxide dismutase (SOD), catalase (CAT), interleukin-6 (IL-6) and tumor necrosis factor-α (TNF-α) in the liver homogenates. G6PC ELISA kit (#MBS097902 MyBioSource, CA, USA), FBP1 ELISA kit (#MBS931493, MyBioSource, CA, USA), and GLY ELISA kit (#MBS1600418; MyBioSource, CA, USA) were used to measure the levels of glucose-6 phosphatase (G-6-Pase), fructose-1,6-bisphosphatase 1 (PBP1), and glycogen in the liver homogenates. The TransAM Nrf2, NF-E2-related factor 2 (Nrf2 DNA binding ELISA) kit (#50296, ActiveMotif, Tokyo, Japan) and TransAM NF-κB p65 ELISA kits (#40096, ActiveMotif, Tokyo, Japan) were used to measure the total and cytoplasmic levels of Nrf2 and NF-κB p65 in the homogenates. All procedures were conducted according to each manufacturer’s instructions for 8 samples/groups.

### 2.10. Real-Time PCR (qPCR)

Real-time PCR was conducted to measure the mRNA transcript levels of Keap-1, NF-κB, Nrf2, and β-actin (a reference gene). All primer sequences were designed and provided by ThermoFisher, Waltham, MA, USA (Table 1). The total RNA was isolated from 0.1 g frozen samples using 1 ml TRIzol reagent (#15596026, Invitrogen, Waltham, MA, USA) as per the manufacturer’s instruction. The first-strand cDNA was synthesized using a commercial kit (#GE27-9261-01, Roche Diagnostic Company, Indianapolis, IN, USA) as per the kit instruction. All amplifications were carried out using a CFX69 real-time PCR system (Biorad, Hercules, CA, USA) according to the amplification steps provided with the SsoFast EverGreen Master Mix kit (#172-5200, Biorad, CA, USA). In brief, the total amplification volume was 20 µL/well, containing the following ingredients: 2 μL cDNA (50 ng/well); 10 µL of the master mix reagent; 0.2 µL of the forward primer (500 nM/each), 0.2 µL of the reverse primer (500 nM/each); and 7.6 µL nuclease-free water. The steps of the amplification were: heating (1 cycle/98 °C/30 s), denaturation (40 cycles/98 °C/5 s), annealing (40 cycles/60 °C/5 s), and melting (1 cycle/5 s/60–95 °C). The relative expression of all targets was normalized to the expression of the reference gene, β-actin.

### 2.11. Hematoxylin and Eosin (H&E) Staining

Formalin-preserved livers were deparaffinized in xylene with reduced levels of 100%, 90%, and 70%. After this, all samples were embedded in wax, cut in a rotatory microtome (3–5 µM), and stained with Harris hematoxylin (H)/glacial acetic acid solution. Next, the samples were de-stained with 1:400 *v/v* HCL/ethanol (70%) solution and stained with one drop of eosin (E). All samples were then dehydrated with ethanol and xylene and covered with mounting media and a coverslip. The next day, all tissues were examined under a light microscope and photographed at 200×.

### 2.12. Statistical Analysis

GraphPad Prism (v. 8) analytic software was used for the statistical analysis of all data. The Kolmogorov–Smirnov test was utilized to test the normality. Analyses were performed using the one-way ANOVA test. The levels of significance were determined using Tukey’s post hoc test (*p* < 0.05). All data were expressed in the results as means ± standard deviation (SD).

## 3. Results

### 3.1. Changes in Rat Body Weights

Final body weights were not significantly changed between the control and the phloretamide-treated rats for either dose (100 and 200 mg/kg) (Table 2). However, final body weights were significantly reduced in the STZ-diabetic rats compared to the control rats (Table 2). Body weights were significantly increased again in both the STZ + phloretamide (100 mg/kg) and STZ + phloretamide (200 mg/kg) rats compared to the STZ-diabetic rats (Table 2). The increment in body weights in the STZ + phloretamide (200 mg/kg)-treated rats was significant compared to the STZ + phloretamide (100 mg/kg)-treated rats, at levels which did not significantly vary from those of the control rats (Table 2).

### 3.2. Changes in the Markers of Glucose Homeostasis

While the levels of fasting insulin were not significantly changed, fasting glucose and hepatic levels of G-6-Pase and FBP-1 were significantly reduced, and hepatic levels of hexokinase and glycogen were significantly increased in the control rats that were administered phloretamide at doses of 100 and 200 mg/kg (Table 2). On the other hand, levels of fasting insulin and hepatic hexokinase and glycogen were significantly decreased, but fasting glucose and hepatic levels of G-6-Pase and FBP-1 were significantly increased in STZ-diabetic rats compared to control and phloretamide (100 and 200 mg/kg)-treated rats (Table 2). These descriptors were significantly reversed in both the STZ + phloretamide (100 mg/kg) and STZ + phloretamide (200 mg/kg) groups compared to the STZ-diabetic rats (Table 2). In the control and STZ-diabetic rats, the effect of phloretamide on all of these markers was significantly more profound with the higher dose (200 mg/kg) compared to those observed with the lower 100 mg/kg dose (Table 2). Despite this, fasting glucose levels and hepatic levels of G-6-Pase and FBP-1 remained significantly higher, while the levels of fasting insulin, the hepatic levels of fructokinase, and the hepatic levels of glycogen in the STZ + phloretamide (200 mg/kg) group remained significantly varied, with the corresponding basal levels observed in the control rats (Table 2).

### 3.3. Histology of the Pancreas

**Figure 1:**
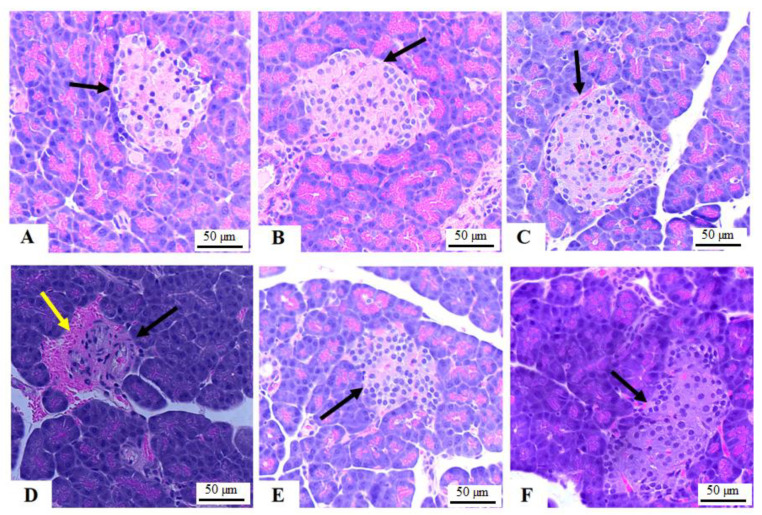
Photomicrographs of the pancreases from all groups of rats as stained by hematoxylin and eosin (H &E) stain: (**A**–**C**): represents control, phloretamide (100 mg/kg)-, and phloretamide (200 mg/kg)-treated rats, and they show normally sized, circular islets of Langerhans containing α-cells and β-cells. (**D**): represents a rat with STZ-induced DM and shows obvious shrinkage in the islets of Langerhans (black arrow) with increased hemorrhaging around the islets (yellow arrow). This pancreas also showed a reduction in the number of cells. (**E**,**F**): represent STZ + phloretamide (100 mg/kg)- and STZ + phloretamide (100 mg/kg)-treated rats, respectively, and they show an increase in the size of the islets of Langerhans (black arrows) and their number of pancreatic α and β-cells. Note that the effect was more profound with a dose of 200 mg/kg (**F**). Magnification = 200×.

Pancreatic tissues obtained from the control, phloretamide (100 mg/kg)-treated, and phloretamide (200 mg/kg)-treated rats showed normally sized and round islets of Langerhans with abundant cell number. Pancreases obtained from STZ-diabetic rats showed shrinkage in the islets of Langerhans with reduced cell number and hemorrhage surrounding the islets (Figure 1D). An increment in the size of the islets as well as in the numbers of their cell content was observed in both the STZ + phloretamide (100 mg/kg)- and STZ + phloretamide (200 mg/kg)-treated rats (Figure 1E,F, respectively). The improvement in the structure of the pancreas was more obvious with the dose of 200 mg/kg.

### 3.4. Changes in the Serum and Hepatic Lipid Profiles

Among all measured lipid-related parameters, treating the rats with phloretamide (100 and 200 mg/kg) resulted in a dose-dependent reduction in the serum levels of FFAs compared to the control rats (Table 3). The serum levels of FFAs, TGs, CHOL, and LDL-c, as well as the hepatic levels of TGs and CHOL were significantly increased, whereas serum levels of HDL-c were significantly reduced in the STZ-diabetic rats compared to the control or phloretamide (100 and 200 mg/kg)-treated rats (Table 3). An opposite picture is seen concerning all of these serum and liver lipid markers in the STZ + phloretamide (100 mg/kg)- and STZ + phloretamide (200 mg/kg)-treated rats compared to the STZ-treated rats (Table 3), an effect that was dose-dependent. Of note, serum and hepatic levels of TGs and CHOL and serum levels of LDL and FFAs remained significantly higher, and serum levels of HDL-c remained significantly lower in the STZ + phloretamide (200 mg/kg)-treated rats compared to the control rats (Table 3).

### 3.5. Changes in the Hepatic Markers of Oxidative Stress and Inflammation

**Figure 2:**
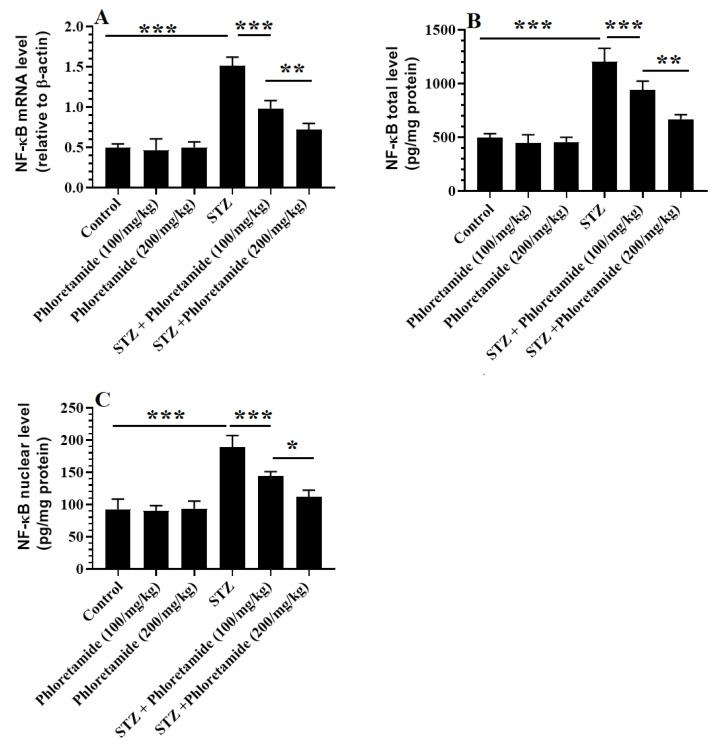
mRNA of NF-κB (**A**) and total and nuclear levels of NF-κB p65 (**B**,**C**) in the livers of all groups of rats. Data are expressed as mean ± SD for *n* = 8 rats/group. Levels of significance at *p* < 0.05 (*), 0.01 (**), or 0.001 (***).

Hepatic levels of TNF-α and IL-6, as well as the mRNA of NF-κB, and total and nuclear levels of NF-κB p65 did not significantly differ between the control, phloretamide (100 mg/kg), and phloretamide (200 mg/kg)-treated rats, but they were significantly higher in the STZ-diabetic rats (Table 4 and Figure 2). In addition, the levels of MDA were significantly higher, but the levels of GSH, SOD, CAT, and HO-1 were significantly lower in the livers of the STZ-diabetic rats compared to the control and phloretamide (100 and 200 mg/kg)-treated rats (Table 4). The levels of MDA, TNF-α, IL-6, NF-κB mRNA, and total and nuclear levels of NF-κB p65 were significantly reduced, but levels of GSH, SOD, CAT, and HO-1 were significantly increased in the livers of both the control and STZ-diabetic rats which received phloretamide at either dose (100 and 200 mg/kg) compared to the control and diabetic rats to which we administered only the vehicle, respectively (Table 4 and Figure 2). However, the levels of all of the biochemical endpoints were improved with the higher dose of the drug, and those levels did not significantly return to their basal levels (Table 4 and Figure 2).

### 3.6. Changes in the Hepatic Keap-1/Nrf2 Axis

**Figure 3:**
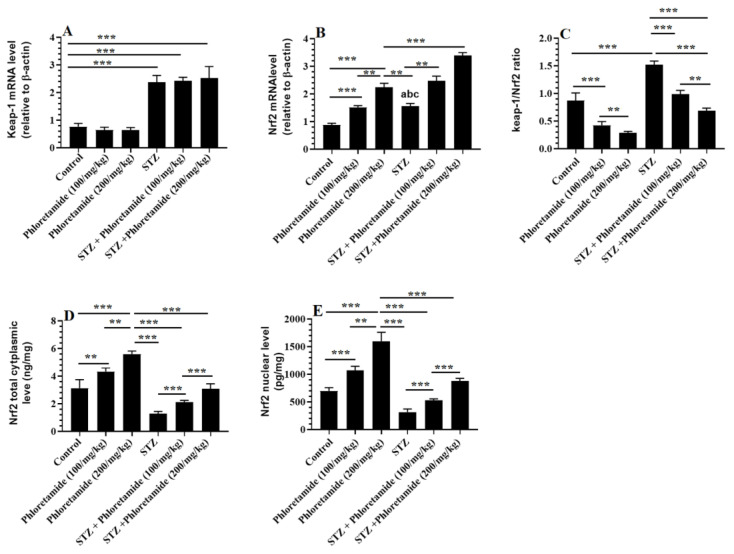
mRNA of Keap-1 and Nrf2 (**A**,**B**), the ratio of mRNA of Keap-1/mRNA of Nrf2 (**C**), and total cytoplasmic and protein levels of Nrf2 (**D**,**E**) in the livers of all groups of rats. Data are expressed as mean ± SD for *n* = 8 rats/group. Levels of significance at *p* < 0.01 (**), or 0.001 (***).

The mRNA of both Keap-1 and Nrf2, as well as the ratios of Keap-1/Nrf2, were significantly increased, whereas total and nuclear levels of Nrf2 were significantly depleted in the livers of the STZ-diabetic rats compared to the control rats (Figure 3A–E). The mRNA levels of Keap-1 did not significantly vary between the control, phloretamide (100 mg/kg)-, and phloretamide (200 mg/kg)-treated rats (Figure 3A). Along those same lines, the mRNA levels of Keap-1 did not significantly vary between the STZ-diabetic, STZ + phloretamide (100 mg/kg), and STZ + phloretamide (200 mg/kg)-treated rats (Figure 3A). The mRNA levels, total, and nuclear levels of Nrf2 were significantly increased, whereas the ratios of Keap-1/Nrf2 were significantly reduced in both the control and the STZ-diabetic rats that were treated with either dose of phloretamide (100 or 200 mg/kg) compared to either the control or the STZ-diabetic rats that were administered only the vehicle (Figure 3B–E). Interestingly, and in both cases, the stimulatory effects of phloretamide on the mRNA, nuclear, and total levels of Nrf2 were higher in the STZ + phloretamide (200 mg/kg)-treated rats compared to those effects afforded by the lower dose (100 mg/kg).

### 3.7. Histological Findings for the Livers

**Figure 4:**
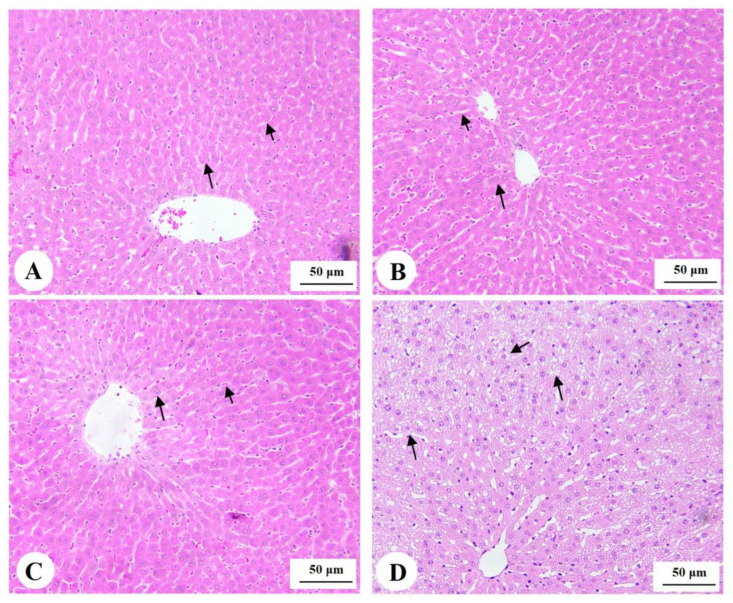
Photomicrographs of histological liver sections stained with hematoxylin and eosin (H&E): (**A**–**C**): from control, phloretamide (200 mg/kg)-treated rats, and phloretamide (200 mg/kg)-treated rats, showing normal liver structures, including central vein (CV), hepatocytes (long arrows), and sinusoids (short arrows). (**D**): from an STZ-diabetic rat, showing increased cytoplasmic fat droplet accumulation of medium and large sizes (long and short arrows, respectively). Magnification = 200×.

**Figure 5:**
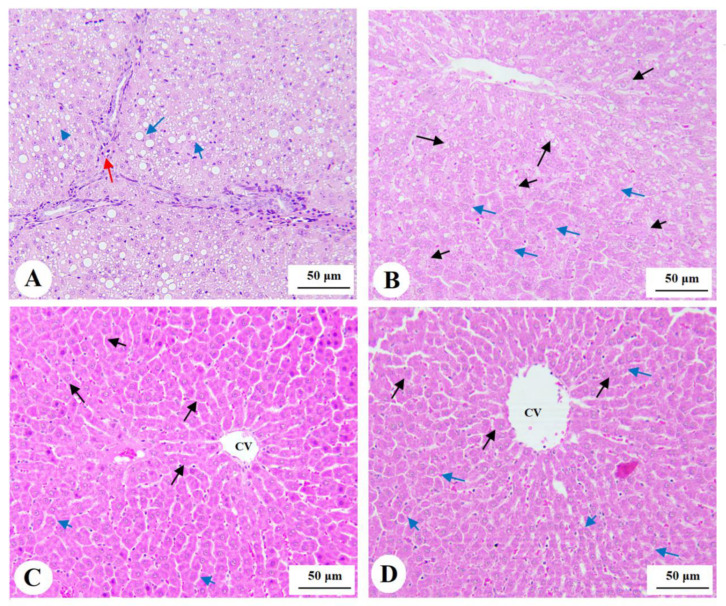
Photomicrographs of histological liver sections stained with hematoxylin and eosin (H&E). (**A**): taken from an STZ-diabetic rat (separate from that shown in Figure 4D), showing increased cytoplasmic fat droplet accumulation of medium and large sizes (long and short blue arrows, respectively), with increased immune cell infiltration (red arrow). (**B**): taken from an STZ + phloretamide (100 mg/kg)-treated rat, showing small improvement in the structure of the hepatocytes and an obvious reduction in the number of hepatocytes containing fat vacuoles compared to the STZ-induced rats, with an increased number of normal cells (blue arrows). However, many cells still contain large- or medium-sized fat vacuoles in their cytoplasm (long and short black arrows). (**C**,**D**): taken from STZ + phloretamide (200 mg/kg)-treated rats, showing the greatest improvement in the liver structure, with almost normal hepatocytes and lacking cytoplasmic fat deposits (long, black arrows). These livers also show normally sized sinusoids (short, blue arrows). However, some damaged cells are still seen (long, blue arrows).

Livers obtained from the control, phloretamide (100 mg/kg)-, and phloretamide (200 mg/kg)-treated rats showed normal histological features, including central veins, sinusoids, and hepatocytes (Figure 4A–C). However, livers obtained from the STZ-diabetic rats showed increased cytoplasmic vacuolation of small, medium, and large size; dilated sinusoids; and immune cell infiltration (Figure 4D and Figure 5A). On the other hand, the livers of the STZ + phloretamide (100 mg/kg)-treated rats showed an improvement in the number of normal hepatocytes, but they still contained a large number of cells showing moderately sized fat vacuoles (Figure 4B). Almost-normal hepatic structures with almost no cytoplasmic vacuoles were seen in the livers of the STZ + phloretamide (100 mg/kg)-treated rats. Nonetheless, some damaged hepatocytes were still seen in this group of rats (Figure 5C,D).

## 4. Discussion

Data from this study revealed a dose-dependent potential of phloretamide to alleviate pancreatic damage and NAFLD in STZ-induced DM in rats. In this study, treatment with phloretamide not only reduced fasting glucose and HBA1c levels, but also attenuated STZ-mediated pancreatic β-cell damage. In addition, it significantly inhibited the hepatic gluconeogenesis in the livers of STZ-diabetic rats by suppressing several key enzymes such as glucose-6 phosphatase (G-6-Pase) and fructose-1,6-bisphosphatase 1 (PBP1). Moreover, treatment with phloretamide ameliorated dyslipidemia and hepatic DNL in these STZ-diabetic rats that were concomitant with suppressing markers of lipid peroxidation and inflammation. However, the hepatic antioxidant and protective effect of phloretamide seem to be associated with activating the Nrf2/antioxidant axis, as well as suppressing the activation of NF-κB p65. Therefore, the overall protective effect of phloretamide against STZ-induced damage and NAFLD disease includes the regeneration of pancreatic beta cells and hypolipidemic, hypoglycemic, antioxidant, and anti-inflammatory effects.

In this study, we utilized STZ, glucosamine–nitrosourea to induce DM in rats, as discussed by other authors, to promote hyperglycemia and hypoinsulinemia [36,37,38]. In general, STZ is a selective, toxic drug for pancreatic β-cells that promotes hyperglycemia and insulin deficiency by damaging these cells via the increased generation of free radicals, such as superoxide (O_2_^−^), hydrogen peroxide (H_2_O_2_), and nitric oxide (NO), as well as by depleting the pancreatic cell levels of NAD+ and ATP [39]. In addition, STZ-mediated DM is associated with a severe reduction in body mass due to increased muscle wasting and stimulated lipolysis in the adipose tissue [30]. In this study, STZ-treated rats showed a significant reduction in their body weights that coincided with a significant increase in serum levels of FFAs, indicating active adipose tissue lipolysis and muscle loss. In addition, their pancreas showed increased shrinkage and the loss of pancreatic β-cells, as well as fasting hypoinsulinemia, thus confirming the pro-oxidant damaging effect of STZ and its role in the development of the observed fasting hyperglycemia.

On the other hand, treatment with phloretamide significantly improved rat body weights and reduced fasting glucose levels in both the control and STZ-diabetic rats in a dose-dependent manner. However, while it failed to modulate insulin levels in control rats, phloretamide slightly but significantly raised circulatory insulin levels in STZ-diabetic rats to levels that remained significantly lower than the basal levels observed in the control rats. These data indicate a potent hypoglycemic effect of phloretamide, which seems to be caused partially by stimulating insulin release from the damaged pancreatic cells, but it is also largely mediated by other mechanisms, such as improving peripheral and hepatic insulin action and regulating hepatic glucose homeostasis. Yet, such an increase in insulin levels in STZ-diabetic rats after phloretamide treatment could be explained by the antioxidant potential that other researchers have identified. However, we did not study the effect of phloretamide on pancreatic markers of oxidative stress to confirm this [27].

To further examine the potential hypoglycemic effect of phloretamide, we targeted key hepatic enzymes responsible for glucose homeostasis. In the liver, glucose stimulates glycogen synthesis and inhibits gluconeogenesis [40]. G-6-Pas and FBP-1 are key enzymes responsible for gluconeogenesis, whereas hexokinase is responsible for glucose degradation [41,42]. Glycogen stores are significantly depleted in the muscles and livers of T1DM animals and humans due to a lack of insulin [43,44,45,46]. This was also documented in the livers of the diabetic rats in this study, which also showed lower stores of hepatic glycogen. In addition, these rats had reduced hepatic hexokinase levels and showed a significant increment in G-6-Pase and FBP-1, which can also be attributed to insulin deficiency/action on the liver, and it explains such a reduction in glycogen stores in these diabetic rats. These data are also supported by the findings of many other authors [36,47]. However, phloretamide not only significantly reversed these events in the STZ-treated rats, but it also modulated the levels of these metabolic enzymes in the same way and in a dose-dependent manner in the livers of the control rats, too. As discussed above, and given that phloretamide did not affect insulin levels in the control rats but significantly reduced circulatory levels of FFAs, these data suggest that phloretamide suppresses hyperglycemia, mainly by regenerating the pancreatic-beta-cells, regulating hepatic glucose hemostasis and by increasing insulin sensitivity and signaling.

Phloretamide is a metabolite that is produced from the metabolism of phloretin and its product, phloretic acid [27]. Although ours is the first experiment to describe this effect for phloretamide, the previously reported hypoglycemic effect of phloretin was attributed to its ability to stimulate the generation of β-cells, increase insulin release, improve peripheral insulin action, stimulate hepatic glycogen synthesis, suppress gluconeogenic enzymes, and upregulate hexokinase in the liver [24,25]. Hence, our data may suggest that phloretin produces its hypoglycemic effect indirectly by generating phloretamide. These data can be also comparable to other previous studies. Indeed, several plant herbal extracts and plant-derived compounds such as salidroside, puerarin, ginseng, vitexin, saponins, and geniposide attenuated fasting hyperglycemia and complications of DM by their potential to attenuate the oxidative damage of the pancreas and promoting regeneration [48]. On the other hand, other plant-derived compounds, fisetin, quercetin, morin, isoleucine, berberine, and berberrubine attenuated hyperglycemia in diabetic and obese animals by improving insulin sensitivity and/or inhibiting hepatic gluconeogenesis [49,50,51].

On the other hand, hyperlipidemia is the major hallmark of DM and NAFLD and occurs mainly due to lipotoxicity and increased influx of FFA from the impaired adipose tissue [5,6,52]. Statins remain the major golden therapy to treat hyperlipidemia in patients with DM and NAFLD [53]. Currently, several plant-derived flavonoids have been also shown to exert anti-diabetic effects and were able to attenuate hyperlipidemia and hepatic steatosis either directly by suppressing de novo lipogenesis or indirectly through increasing insulin sensitivity and adipose tissue lipogenesis [54]. Examples include quercetin, rutin, kaempferol, isorhamnetin, fisetin, hesperidin, naringenin, eriodictyol, curcumin, and apigenin [54]. In this examination, we have detected a significant increase in TGs, CHOL, FFAs, and LDL-c in the serum of STZ-treated rats, coinciding with low serum levels of HDL-c and higher levels of CHOL and TGs in their livers. In addition, the livers of the STZ-diabetic rats showed severe damage and hepatocyte ballooning, indicating the progression toward NASH. These data also support many other authors who have also shown similar results [55,56]. On the contrary, phloretamide, at both 100 and 200 mg/kg, significantly attenuated hyperlipidemia and hepatic steatosis in a dose-dependent manner in the STZ-diabetic rats. However, it failed to modulate the levels of all of these lipids in the serum and livers of the control rats, even at the higher dose. These data suggest that the hypolipidemic effect of phloretamide is not direct and is probably secondary due to its amelioration of fasting hyperglycemia. Yet, further examination is required to confirm this. However, the hypolipidemic effect of phloretin has been reported in the literature, where it was attributed to suppressing DNL via the activation of SIRT1/AMPK signaling, which can inhibit SREBP1c [57].

According to the multiple-hit hypothesis, oxidative stress and inflammation are the primary mechanisms that lead to hepatic damage and the progression to NASH in diabetic conditions [58]. Higher levels of oxidative stress and inflammation markers, as well as reduced levels of antioxidants, are common in the livers of animals and patients with NAFLD [19]. On the one hand, the increase in the metabolism of FFAs and the subsequent impairment in the process of oxidative phosphorylation, mitochondrial damage, and ER stress are the major pro-oxidant pathways that generate ROS in the livers of diabetic animals [3,4,8]. In addition, high glucose levels can promote liver damage, fibrosis, and apoptosis by generating high levels of ROS through several mechanisms, including auto-oxidation and the activation of the PKC, polyol, hexose amine, and advanced glycation end-product (AGE) pathways [8,30]. In addition, ROS and NF-κB are positively crossed with each other during tissue damage and can stimulate each other in a vicious cycle.

Similar to these data, MDA, TNF-α, and IL-6 levels were significantly increased, while levels of the measured antioxidants (i.e., GSH, SOD, and CAT) were significantly reduced in the livers of the STZ-diabetic rats in this study. In addition, the STZ-treated rats showed a significant increase in the mRNA levels of NF-κB, as well as in the total and nuclear levels of NF-κB p65, which may explain the source of the increase in the levels of ROS. These findings are similar to those of many other authors [30,59,60]. On the contrary, these alterations were reversed by both dosage levels of phloretamide, with more profound results to be seen with the higher dose, thus illustrating the antioxidant and anti-inflammatory effects of this compound and supporting the previously reported hepatic antioxidant potential of phloretamide in vitro.

Although these effects could be attributed to the improvement in glucose and FFA metabolism in diabetic rats post administration of phloretamide, phloretamide treatment in the control rats also reduced levels of MDA and increased the content of all measured antioxidant enzymes in a dose-dependent manner. Since no changes in the levels of TNF-α and IL-6, nor in the expression, total, or nuclear levels of NF-κB were observed in the livers of the control rats to which we administered phloretamide, it seems very reasonable that the silencing of the inflammatory response and the activation of NF-κB is mediated by the glucose-independent antioxidant potential of phloretamide. Supporting this, and independent of any other pathway, treating normally cultured hepatocytes with a physiological dose of phloretamide stimulated levels of glutathione S-transferase (GST) isoenzymes, NAD(P)H: quinone oxidoreductase-1 (NQO1), and heme-oxygenase-1 (HO-1) [27]. In addition, drugs that activated antioxidants or treatment with N-acetyl cysteine (NAC) attenuated hepatic inflammation by suppressing NF-κB [61,62]. Additionally, antioxidant therapy attenuated NAFLD in diabetic rats by suppressing NF-κB [30].

Nonetheless, the activation of Nrf2 is novel therapy to treat NAFLD by upregulating antioxidants, suppressing NF-κB, and regulating the activities of some genes involved in DNL and FA mitochondrial oxidation [18,19]. Associated with all of the abovementioned changes in the livers of the diabetic rats, there was a significant increment in the hepatic expression of both Keap-1 and Nrf2 in the livers of the STZ-treated rats. This was expected due to the role of ROS in upregulating Keap-1 and Nrf2 [19]. However, the ratio of Keap-1/Nrf2 in the livers of these diabetic rats was almost 1.5 times higher than the ratio in the control rats (<1). Such a higher ratio of Keap-1/Nrf2 may explain why the livers of these diabetic rats showed a significant reduction in the nuclear levels of Nrf2 despite the increment in its transcription. However, the significant progressive reduction in the expression ratio of Keap-1/Nrf2 after increasing the doses of the phloretamide treatment explains why the livers of these treated rats showed higher nuclear levels of NrfA, similar to the reduction in the activation of Nrf2 in the livers of STZ-diabetic rodents, with or without NAFLD [9,10,11]. Therefore, it seems reasonable that the reduced activation of Nrf2 is a key mechanism for developing hepatic steatosis and oxidative stress in the livers of these rats. In support of this theory, Nrf2-deficient rats that were fed a methionine- and choline-deficient (MCD) diet showed accelerated lipid accumulation, reduced levels of GSH and antioxidant enzymes, and increased expression of cytochrome P450 enzymes, with normal glucose metabolism compared to Nrf2+/+ rats fed the same diet [12,13].

On the other hand, several plant extracts and flavonoids also attenuated diabetic complications and prevented several tissue injuries in other animal models by activating the Nrf2/antioxidant axis [22]. The most interesting finding observed in this study is that treatment with phloretamide also increased the transcription of Nrf2 and stimulated its nuclear levels in a dose-dependent manner in both the control and diabetic rats, thus suggesting a regulatory role for this factor, irrespective of circular glucose levels. This effect was determined to be independent of modulations in the expression of Keap-1, as similar mRNA levels of Keap-1 were observed between the diabetic rats, with or without phloretamide treatment. Based on this observation in the model rats, we can conclude that phloretamide might attenuate hepatic steatoses and oxidative stress via its stimulatory role on Nrf2, and this pathway seems to be a major mechanism of action. Indeed, several plant flavonoids can upregulate Nrf2 in a ROS-independent mechanism by regulating their transcription and translation [22]. Similar to our findings, restoring Nrf2 in MCD mice alleviated the hepatic phenotype toward a reduction in hepatic steatosis, mainly by activating PPARα and suppressing SREBP1 [13]. Additionally, treating diabetic rats with phloretin attenuated diabetic cardiomyopathy by inhibiting the interaction between Nrf2 and Keap-1, independent of its hypoglycemic effect [63]. Moreover, phloretin attenuated several types of organ damage in other non-diabetic animal models by activating Nrf2/antioxidant/NF-κB signaling [23,24,25,26].

Although these data seem very interesting, they are still observations and have some limitations. Importantly, whether Nrf2 is the upstream mechanism of action of phloretamide that regulates oxidative stress, antioxidant levels, DNL, and the activity of NF-κB cannot be concluded based solely on these data. Therefore, it is highly advisable to repeat this work in cell cultures or animals lacking Nrf. This will enable us to further draw a direct conclusion.

### Study Limitations

Despite these findings, the above current study still has some critical limitations. On the one hand, the basis of selection of the doses of the phloretamide was based on their hypoglycemic effect in our pilot studies. Until now, no studies have been available to show the optimum concentration of naturally produced phloretamide that can be found in vivo as the metabolite from phloretine. Hence, a more comprehensive approach targeting pharmacokinetics, absorption, availability, and tissue levels of phloretamide should be considered to minimize the dose of treatment. In addition, our data are still observational. Therefore, more well-designed studies using animals deficient with Nrf2 are highly recommended for a better understanding of the mechanism of action of this flavonoid. Furthermore, molecular mechanisms responsible for the hypoglycemic and hypolipidemic effect of phloretamide such as its effect on key marker genes should be examined in both the liver and adipose tissue to widen our knowledge about the action of phloretamide.

## 5. Conclusions

The findings of this study are the first in the literature to demonstrate the potential to use phloretamide as a future “golden drug” to treat hyperglycemia-induced diabetic complications and NAFLD due to its ability to regulate oxidative stress and inflammation. These findings are an encouragement to further test these molecules in preclinical and clinical trials in diabetic patients, as well as in other disorders where the deficiency of Nrf2 is a key mediator.

## Figures and Tables

**Table 1:** Characteristics of the primers for real-time PCR.

Gene	Primers (5′→3′)	Accession	BP
Nrf2	F:-AAAATCATTAACCTCCCTGTTGAT R: R:′-CGGCGACTTTATTCTTACCTCTC	NM_031789	118
NF-κB	F: GTGCAGAAAGAAGACATTGAGGTG R: AGGCTAGGGTCAGCGTATGG	XM_342346.4	176
Β-actin	F: GACCTCTATGCCAACACAGT R: CACCAATCCACACAGAGTAC	NM_031144	154

**Table 2:** Changes in body weight and selected markers of glucose homeostasis in all groups of rats.

	Control	Phloretamide (100 mg/kg)	Phloretamide (200 mg/kg)	STZ	STZ + Phloretamide (100 mg/kg)	STZ + Phloretamide (200 mg/kg)
Final body weight (g)	482 ± 32	471 ± 41	489 ± 54	382 ± 29	431 ± 32 ^abcd^	473 ± 36 ^de^
Fasting glucose (mg/dL)	122 ± 8.1	107.7 ± 8.5 ^a^	91.5 ± 7.9 ^ab^	354 ± 29 ^abc^	235 ± 15.7 ^abcd^	153 ± 11.6 ^abcde^
Fasting insulin (ng/mL)	4.5 ± 0.68	5.1 ± 0.43	4.8 ± 0.65	1.2 ± 0.27 ^abc^	1.89 ± 0.32 ^abcd^	2.68 ± 0.34 ^abcde^
Hepatic Hexokinase (pg/mL)	18.6 ± 1.3	22.4 ± 1.8 ^a^	39.5 ± 3.3 ^ab^	6.7 ± 0.82 ^abc^	9.2 ± 1.5 ^acbd^	14.5 ± 2.1 ^abcde^
Hepatic G-6-Pase (U/mg)	9.8 ± 0.79	6.8 ± 0.73 ^a^	4.6 ± 0.53 ^ab^	22.7 ± 2.9 ^abc^	16.7 ± 2.1 ^acbd^	12.4 ± 1.1 ^abcde^
Hepatic FBP-1 (pg/mg)	114 ± 7.6	91.6 ± 7.1 ^a^	81.8 ± 6.8 ^ab^	422 ± 32 ^abc^	312 ± 27 ^acbd^	178 ± 14.7 ^abcde^
Liver glycogen (mg/mg)	35.7 ± 2.4	48.8 ± 5.6 ^a^	61.5 ± 6.8 ^ab^	17.6 ± 2.1 ^abc^	24.7± 12.5 ^acbd^	29.1 ± 1.9 ^abcde^

Data are expressed as mean ± SD for *n* = 8 rats/group. Levels of significance at *p* < 0.05: ^a^: vs. control; ^b^: vs. phloretamide (100 mg/kg)-treated rats; ^c^: vs. phloretamide (200 mg/kg)-treated rats; ^d^: vs. STZ-diabetic rats, ^e^: vs. STZ + phloretamide (100 mg/kg)-treated rats.

**Table 3:** Serum and hepatic lipid profiles of all groups of rats.

		Control	Phloretamide (100 mg/kg)	Phloretamide (200 mg/kg)	STZ	STZ + Phloretamide (100 mg/kg)	STZ + Phloretamide (200 mg/kg)
Serum	TGs (mg/dL)	67.5 ± 5.4	61.7 ± 7.1	68.9 ± 6.5	182 ± 15.6	124.5 ± 12.3 ^abcd^	101 ± 8.7 ^abcde^
	CHOL (mg/dL)	74.5 ± 6.5	75.1 ± 5.8	69.9 ± 6.3	207 ± 17.8 ^abc^	141 ± 11.3 ^abcd^	97.6 ± 8.7 ^abcde^
	LDL-c (mg/dL)	42.5 ± 4.1	38.9 ± 5.1	44.7 ± 5.9	147 ± 9.7 ^abc^	87.6 ± 7.5 ^abcd^	64.5 ± 5.9 ^abcde^
	HDL-c (mg/dL)	19.8 ± 1.9	22.4 ± 2.1	21.7 ± 2.4	8.7 ± 1.2 ^abc^	12.3 ± 1.3 ^abcd^	17.8 ± 1.1 ^abcde^
	FFAs (µmol/L)	445 ± 37	389 ± 25.6 ^a^	301± 25.8 ^ab^	988 ± 76.8 ^abc^	763 ± 65.8 ^abcde^	578 ± 61.3 ^abcde^
Liver	Triglycerides (mg/g)	4.23 ± 0.35	3.83 ± 0.59	4.45 ± 0.65	7.64 ± 0.69 ^abc^	6.18 ± 0.72 ^abcd^	5.35 ± 0.54 ^abcde^
	CHOL (µg/g)	2.56 ± 0.39	2.6 ± 0.7	2.77 ± 0.64	6.89 ± 0.45 ^abc^	5.13 ± 0.68 ^abcd^	3.38 ± 0.41 ^abcde^

Data are expressed as mean ± SD for *n* = 8 rats/group. Levels of significance at *p* < 0.05: ^a^: vs. control; ^b^: vs. phloretamide (100 mg/kg)-treated rats; ^c^: vs. phloretamide (200 mg/kg)-treated rats; ^d^: vs. STZ-diabetic rats, ^e^: vs. STZ + phloretamide (100 mg/kg)-treated rats.

**Table 4:** Hepatic levels of selected oxidative stress, antioxidant, and inflammatory markers in all groups of rats.

	Control	Phloretamide (100 mg/kg)	Phloretamide (100 mg/kg)	STZ	STZ + Phloretamide (100 mg/kg)	STZ + Phloretamide (200 mg/kg)
MDA (pmol/mg)	567 ± 84	432 ± 37 ^a^	432 ± 37 ^a^	1654 ± 134 ^abc^	924 ± 79 ^abcd^	689 ± 73 ^abcde^
SOD (U/mg)	28.7 ± 2.5	36.7 ± 6.7 ^a^	36.7 ± 6.7 ^a^	13.4 ± 1.7 ^abc^	19.8 ± 2.4 ^abcd^	26.9 ± 2.9 ^bcde^
GSH (µg/mg tissue)	64.6 ± 6.1	75.4 ± 6.8 ^a^	75.4 ± 6.8 ^a^	25.6 ± 1.6 ^abc^	39.5 ± 4.1 ^abcd^	54.3 ± 4.3 ^abcde^
CAT (U/mg tissue)	8.2 ± 1.2	12.4 ± 1.5 ^a^	12.4 ± 1.5 ^a^	3.2 ± 0.56 ^abc^	7.8 ± 0.82 ^cbd^	12.4 ± 1.4 ^abcde^
HO-1 (ng/mg tissue)	5.6 ± 0.45	7.8 ± 0.37 ^a^	7.8 ± 0.37 ^a^	1.34 ± 0.28 ^abc^	3.54 ± 0.49 ^acbd^	5.8 ± 0.54 ^abcde^
IL-6 (pg/mg tissue)	22.4 ± 2.4	25.6 ± 3.4	25.6 ± 3.4	87.6 ± 5.9 ^abc^	51.2± 5.2 ^acbd^	33.7 ± 1.6 ^abcde^
TNF-α (pg/mg tissue)	4.5 ± 0.23	5.1 ± 0.73	5.1 ± 0.73	34.5 ± 2.8 ^abc^	17.6 ± 1.6 ^acbd^	9.4 ± 1.1 ^abcde^

Data are expressed as mean ± SD for *n* = 8 rats/group. Levels of significance at *p* < 0.05: ^a^: vs. control; ^b^: vs. phloretamide (100 mg/kg)-treated rats; ^c^: vs. phloretamide (200 mg/kg)-treated rats; ^d^: vs. STZ-diabetic rats, ^e^: vs. STZ + phloretamide (100 mg/kg)-treated rats.

## Data Availability

The datasets used and analyzed during the current study are available from the corresponding author on reasonable request.
